# Wnt-reporter expression pattern in the mouse intestine during homeostasis

**DOI:** 10.1186/1471-230X-8-57

**Published:** 2008-12-02

**Authors:** Paige S Davies, Adria D Dismuke, Anne E Powell, Kevin H Carroll, Melissa H Wong

**Affiliations:** 1 Department of Dermatology, Oregon Cancer Center, Oregon Stem Cell Center, Oregon Health & Science University, Portland, OR, USA; 2 Department of Molecular and Medical Genetics, Oregon Health & Science University, Portland, OR, USA; 3 Department of Cell and Developmental Biology, Oregon Health & Science University, Portland, OR, USA

## Abstract

**Background:**

The canonical Wnt signaling pathway is a known regulator of cell proliferation during development and maintenance of the intestinal epithelium. Perturbations in this pathway lead to aberrant epithelial proliferation and intestinal cancer. In the mature intestine, proliferation is confined to the relatively quiescent stem cells and the rapidly cycling transient-amplifying cells in the intestinal crypts. Although the Wnt signal is believed to regulate all proliferating intestinal cells, surprisingly, this has not been thoroughly demonstrated. This important determination has implications on intestinal function, especially during epithelial expansion and regeneration, and warrants an extensive characterization of Wnt-activated cells.

**Methods:**

To identify intestinal epithelial cells that actively receive a Wnt signal, we analyzed intestinal Wnt-reporter expression patterns in two different mouse lines using immunohistochemistry, enzymatic activity, *in situ *hybridization and qRT-PCR, then corroborated results with reporter-independent analyses. Wnt-receiving cells were further characterized for co-expression of proliferation markers, putative stem cell markers and cellular differentiation markers using an immunohistochemical approach. Finally, to demonstrate that Wnt-reporter mice have utility in detecting perturbations in intestinal Wnt signaling, the reporter response to gamma-irradiation was examined.

**Results:**

Wnt-activated cells were primarily restricted to the base of the small intestinal and colonic crypts, and were highest in numbers in the proximal small intestine, decreasing in frequency in a gradient toward the large intestine. Interestingly, the majority of the Wnt-reporter-expressing cells did not overlap with the transient-amplifying cell population. Further, while Wnt-activated cells expressed the putative stem cell marker Musashi-1, they did not co-express DCAMKL-1 or cell differentiation markers. Finally, gamma-irradiation stimulated an increase in Wnt-activated intestinal crypt cells.

**Conclusion:**

We show, for the first time, detailed characterization of the intestine from Wnt-reporter mice. Further, our data show that the majority of Wnt-receiving cells reside in the stem cell niche of the crypt base and do not extend into the proliferative transient-amplifying cell population. We also show that the Wnt-reporter mice can be used to detect changes in intestinal epithelial Wnt signaling upon physiologic injury. Our findings have an important impact on understanding the regulation of the intestinal stem cell hierarchy during homeostasis and in disease states.

## Background

It is well established that the canonical Wnt signaling pathway plays a critical role in regulating intestinal proliferation at the level of the stem cell [1-6] and has been inferred to regulate proliferation of all intestinal crypt-based cells including the bulk of proliferative cells, the transient-amplifying-cell (TA-cell) population [1-7]. Surprisingly, the proliferative influence of the Wnt signal on discrete cell populations within the crypt has not been previously characterized. Confounding issues for making these distinctions is that manipulation of Wnt signaling in the stem cell population will invariably affect the downstream TA-cell population, complicating interpretation. Further, there is precedence for a Wnt signal acting as a global regulator of proliferation in development prior to the establishment of the stem cell hierarchy[1]. However, there is also evidence that proliferative control of crypt-based cells may be more multi-faceted than originally thought. Most interestingly, the TA-cell population does not express the recently identified Wnt-target stem cell marker, Lgr5[8], nor does it harbor nuclear β-catenin staining, a hallmark of activated Wnt signaling[9,10]. In addition, Wnt signaling has been shown to differentially regulate stem cell and TA-cell populations in other epithelial systems such as the skin[11,12], suggesting that a more complex regulation of proliferation may exist. Therefore, determining the influential distinction of the Wnt signal within the different proliferative intestinal cell populations is important for understanding epithelial homeostasis, regeneration after injury, and cellular dynamics during proliferative diseases.

Epithelial proliferation is confined to the intestinal crypts. The proliferative capacity of the intestine is defined by approximately 4–6 active stem cells and a second rapidly proliferating crypt population made up of the TA-cells that is situated adjacent to the stem cells. Multiple signaling cascades, including the Wnt, Notch, and Sonic Hedgehog pathways[13], converge within the crypt niche to regulate the gradient of proliferation-to-differentiation. The canonical Wnt signaling pathway is well established as an important regulator of intestinal epithelial proliferation[1] and homeostasis[1,14-16]. During mouse intestinal development, ablation of the downstream transcription factor, Tcf4 links loss of Wnt signaling with a loss of epithelial proliferation[1]. In the adult mouse, a proliferative role for this pathway is recapitulated when the Wnt inhibitor Dickkopf-1 is over-expressed, leading to collapse of the crypt structure[2], and most notably in disease, where mutations in this pathway result in epithelial hyperproliferation leading to colorectal cancer[5].

The canonical Wnt signal is conveyed through the binding of a soluble ligand to cell surface co-receptors, Frizzled and Lrp5/6[17], then propagated by inhibiting the degradation of β-catenin, which stimulates the transcription of target genes[18,19]. The Wnt target gene Lgr5 is a putative stem cell marker based upon its crypt mRNA localization and a functional knock-in reporter experiment[8]. Interestingly, Lgr5 is expressed only in epithelial columnar cells, but not higher up in the crypt within the TA-cell population. This suggests that Wnt signals may influence discrete cell populations rather than act as a global proliferative regulator within the crypt. Therefore, it is possible that proliferation of stem cells and the TA-cell population are differentially controlled.

In other systems, such as the hematopoietic system, the Wnt signal also provides proliferative cues to progenitor cells[20]. Self-renewal of both the hematopoietic stem cells and their TA-cell populations are thought to be regulated by the Wnt pathway. Conversely, in epithelial systems such as the skin, stem and TA-cell populations appear to be differentially activated by Wnt signals[11,12]. In the intestine, however, definitive stem cell markers have been slow to be established. The absence of these markers and the inability to accurately distinguish stem and progenitor populations within the intestinal crypt presents an obstacle for determining if Wnt acts as a global regulator of cell proliferation. One approach to establishing the role of Wnt signaling on the discrete intestinal crypt cell populations is to characterize cells within the crypt that are Wnt-activated. Here, we validate for the first time, the Wnt-reporter mouse as a useful resource for evaluation of Wnt-activation within the intestine. Further, we establish that during intestinal homeostasis, activation of the Wnt pathway occurred primarily in an intestinal progenitor cell and not in the actively cycling TA-cell population. Our data validates the Wnt-reporter mouse as a functional tool for detecting changes in Wnt signaling within the intestinal epithelium. We show that the canonical Wnt pathway is stimulated in response to gamma-irradiation-induced apoptosis both by an increased expression of the Wnt-reporter as well as Wnt ligands and the *c-Myc *target gene. The characterization of Wnt signaling within the intestine provides an important foundation for understanding the regulation of the intestinal stem cell hierarchy during homeostasis and in disease states.

## Methods

### Mice

Mice were housed in a specific pathogen-free environment under strictly controlled light cycle conditions, fed a standard rodent Lab Chow (#5001 PMI Nutrition International, Brentwood, MO), and provided water ad libitum. All procedures were performed in accordance to the OHSU Animal Care and Use Committee. The Wnt-reporter TOPGAL[11], C57Bl/6, and Min[21] mice were obtained from The Jackson Laboratory (Bar Harbor, ME) and the BatGal Wnt-reporter mice were a kind gift from Dr. Stefano Piccolo[22].

### Analyses of Wnt-responsive intestinal cells

#### Immunohistochemical analyses

Wnt signaling activity was characterized in adult TOPGAL, BatGal, and C57Bl/6 mouse intestines. The entire length of the intestine was prepared for frozen or paraffin sectioning and the methods used for single and multi-label immunohistochemical staining are previously described[23]. The following antisera were used: anti-β-galactosidase (β-gal; Immunology Consultants Laboratory, Inc.; Newberg, OR; 1:500 dilution), anti-Musashi-1 (#14H-1; a gift from Dr. H. Okano, Keio University, Tokyo; 1:500), UEA-1 (Sigma; St. Louis, MO; 1:500), anti-cryptidin (a kind gift from Andy Oulette, University of California – Irvine; 1:25) and anti-5-HT (Serotonin; Incstar; Stillwater, MN; 1:500). Primary antibodies were detected with species appropriate secondary antibodies conjugated to Cy3, FITC (Jackson ImmunoResearch; West Grove, PA) or Alexa-488 (Molecular Probes; Eugene, OR). Tissues were counterstained with Hoechst (33258; Sigma; St. Louis, MO; 0.1 μg/ml). Paraffin embedded tissue sections were stained with antibodies for β-catenin (Transduction Labs; Lexington, KY; 1:500 dilution) to detect nuclear localization. Staining was performed as described previously[24]. Biotinylated secondary antibodies and Diaminobenzidine (DAB) were employed for visualization. Images were captured on a DMR microscope and DC500 digital camera with IM50 Image Manager Software (Leica Microsystems; Bannockburn, IL). Cy3 images were captured as grayscale and digitally converted to red images.

#### Quantification of β-gal-positive cells

To establish the percentage of β-gal-positive crypts and villi down the length of the intestine, tissue sections from mice stained with antibodies to β-gal as described above were quantified. At least 1500 crypts or villi were screened from n = 2–5 mice and reported as a percentages. For a more detailed analysis of the location of β-gal-positive cells within the crypt, the proximal small intestinal crypts were divided into equal thirds. β-gal-positive cells for each region (upper, middle and lower third) were tallied and compared to the total number of β-gal-positive crypt cells (>1500 crypts; n = 2 mice). To determine the percentage of dual-labeled β-gal-expressing Paneth cells, tissue sections were co-stained with UEA-1 and β-gal antibodies as described above. A total of >1500 crypts/mouse were screened (n = 3 mice).

#### β-gal enzymatic activity

Five micron frozen sections were washed in phosphate buffered saline and prepared for 5-bromo-4-chloro-3-inodyl β-D-galactoside (X-gal) detection followed by nuclear-fast red counterstain modified from previously described protocols[25].

#### Assessment of proliferative status

To detect proliferating cells, 5 μm frozen tissue sections were stained with antibodies against Ki67 (Abcam #ab15580; Cambridge, MA; 1:250) and appropriate fluorescent-conjugated secondary antibodies. Alternatively, mice were injected with 5-bromo-2'deoxyuridine/5-fluoro-2'deoxyuridine (BrdU/FrdU, 120/12 mg/kg body weight; Sigma; St. Louis, MO) 48 h prior to sacrifice. Tissue sections were co-stained sequentially with antibodies to BrdU and β-gal. For BrdU staining a modified protocol from the Abcam Resources website was used . Briefly, tissues were washed in phosphate buffered saline and incubated in blocking buffer (1% BSA, 0.3% Triton X-100, 1 mM CaCl_2_) prior to staining with antibodies to BrdU[23] (a gift from Dr. Jeffrey Gordon, Washington University School of Medicine, St. Louis, MO; 1:1000) and detected with fluorescent secondary antibodies. The tissue was imaged after each step and the acquired images overlayed using Canvas software (ACD Systems; Miami, FL). To quantify β-gal and BrdU expression, >1000 crypts per animal (n = 3) were scored for crypts containing co-labeled cells.

### Wnt signaling response to intestinal damage

#### Irradiation-induced epithelial damage

TOPGAL and C57Bl/6 mice were exposed to 12 Gy and sacrificed at 1, 12, 24, 48, and 72 h post-irradiation. Intestinal tissue was harvested and processed as described above and stained with Hematoxylin & Eosin (H&E) or with antibodies for β-gal or β-catenin. The number of β-gal-positive crypts were counted and compared to the total number of crypts in each tissue section (≥ 1300 crypts counted/time point). Intestinal samples from at least three mice per time point were analyzed (n = 19 mice total). Further, for untreated and 24 h post-irradiation time points, the number of β-gal-positive cells per crypt (1, 2 or >2) was also tallied and normalized to the total number of crypts (>1500 crypts counted/time point; n = 2 mice each). The number of cells co-stained with antibodies for Ki67 and β-gal were also determined for both non-irradiated (n = 2) and 24 h-post-irradiated intestines (n = 2; ≥ 2000 crypts/animal). Average values were represented ± standard deviation. Statistical significance was determined by unpaired *t*-tests assuming equal variances using Microsoft Excel. *p *values < 0.05 were considered significant.

### mRNA expression

#### *In situ *hybridization

To validate the gene expression pattern of *lacZ*, RNA *in situ *hybridization was performed as previously described[26] using digoxigenin-labeled LacZ riboprobes (1 μg/μl), alkaline-phosphatase-conjugated anti-digoxigenin antibody and BM Purple substrate (Roche; Indianapolis, IN).

#### Quantitative analysis of mRNA expression from isolated intestinal cell populations

β-gal mRNA has a shorter half-life than the protein[27,28] and can provide a more precise detection of Wnt-activated cells. A modified Weiser preparation[29,30] was used to isolate crypt and villus epithelium from adult Wnt-reporter mouse small intestine. Differentiated epithelial cells were removed in 1 mM EDTA and 1 mM DTT, where crypt epithelium was isolated in 1 mM EDTA and 5 mM DTT. Total RNA was purified from the isolated villus and crypt cell populations and cDNA was synthesized as we have previously described[31]. Quantitative RT-PCR was performed using a SYBR Green-based assay, primers to β-gal and a 7900 HT Sequence Detector according to established protocols[31,32]. Each cDNA sample was analyzed in triplicate, along with triplicate samples of the endogenous reference gene, Glyceraldehyde-3-phosphate dehydrogenase. Each assay for *lacZ *expression was performed at least three independent times on n = 3 mice. The fold-change was determined using established methods[31,32] and reported relative to levels in crypts.

To demonstrate intestinal Wnt-responsiveness in the TOPGAL model, mice were irradiated as described above and sacrificed 24 h later. Crypt epithelial cells were isolated from the small intestine as described above and evaluated by qRT-PCR for gene expression of three Wnt ligands (*Wnt3*, *Wnt6*, *Wnt9b*), a secreted Wnt inhibitor (*sFrp2*), and a Wnt target gene (*c-Myc*) (n = 2 non-irradiated, n = 3 irradiated). For Wnt9b, only distal small intestinal crypt epithelium was surveyed, due to its restricted expression to this region[33]. Primer sequences are presented in Table 1.

**Table 1:** Primer sequences for qRT-PCR.

**Gene**	**Forward Sequence**	**Reverse Sequence**
*lacZ*	5'-GATCTTCCTGAGGCCGATACTG-3'	5'-GGCGGATTGACCGTAA TGG-3'
*gapdh*	5'-TGGCAAAGTGGA GATTGTTGCC-3'	5'-AAGATGGTGATGGGCTTCCCG-3'
*wnt3*	5'-CAAGCACAACAATGAAGCAGGC-3'	5'-TCGGGACTCACGGTGTTTCTC-3'
*wnt6*	5'-TGCCCGAGGCGCAAGACTG-3'	5'-ATTGCAAACACGAAAGCTGTCTCTC-3'
*wnt9b*	5'-AAGTACAGCACCAAGTTCCTCAGC-3'	5'-GAACAGCACAGGAGCCTGACAC-3'
*sfrp2*	5'-AGGTCCTTTGATGCTGACTGTAAA-3'	5'-TCGGCTTCACCTTTTTGCA-3'
*c-myc*	5'-AGCTTCGAAACTCTGGTGCATAA-3'	5'-GGCTTTGGCATGCATTTTAATT-3'

## Results

### Activation of Wnt signaling in single cells within the intestinal crypt

**Figure 1:**
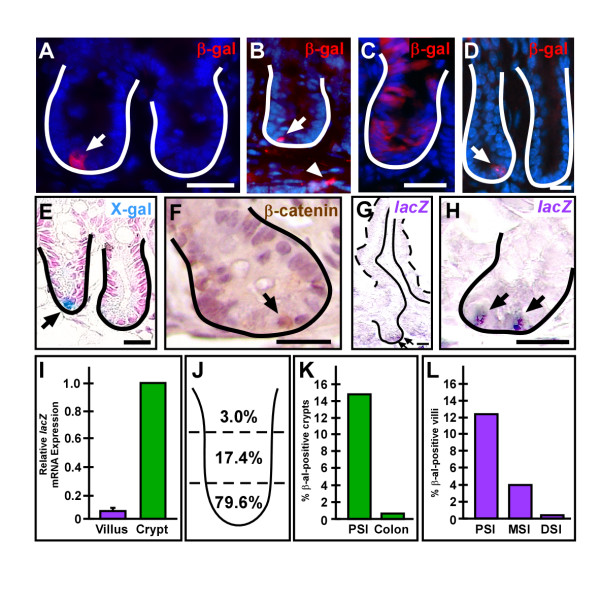
**Adult mouse expression pattern of Wnt-receiving epithelial cells**. (A,C,D) Cryopreserved adult TOPGAL mouse proximal small intestinal (PSI) or colonic and (B) BatGal mouse PSI tissue sections were stained with antibodies against β-galactosidase (β-gal, red) and counterstained with Hoechst dye (blue). (A,B) The majority of crypts in the PSI contained only one Wnt-activated cell or was devoid of positive cells (arrow). There were occasional mesenchymal cells positive for β-gal (arrowhead) in BatGal intestines (B). (C) Occasionally, β-gal-expressing cells were detected throughout the crypt epithelium and on adjacent villi. (D) Colonic crypts contained only rare single β-gal positive cells near the crypt base. (E) Wnt-receiving cells detected by enzymatic activity, X-gal staining (blue, arrow). (F) Adult wild-type mouse PSI was stained with antibodies against β-catenin (brown; arrow) to detect nuclear expression and counterstained with Hematoxylin. (G-I) Analyses of reporter RNA expression pattern and localization was determined by *in situ *hybridization (G,H; purple, arrow) and are consistent with the expression pattern in (A). (I) qRT-PCR for *lacZ *gene expression in isolated crypt or villus epithelial cells from TOPGAL PSI demonstrated expression in the crypts. (J) The crypt localization of β-gal-positive cells was highest in the lower third and decreased in numbers in the middle and upper third. (K) Crypts with Wnt-receiving cells in TOPGAL intestinal sections were higher in the PSI (15.2%) and decreased down the length of the intestine to 0.8% in the colon. (L) The number of β-gal-positive villi also reflected a decreasing gradient with the highest numbers in the PSI (12.3%), less in the the middle small intestine (MSI; 4.0%) and the least in the distal small intestine (DSI; 0.2%). Solid white or black line demarks the epithelial-mesenchymal boundary. Dashed line outlines the apical epithelial border. Bar = 25 μm.

**Figure 2:**
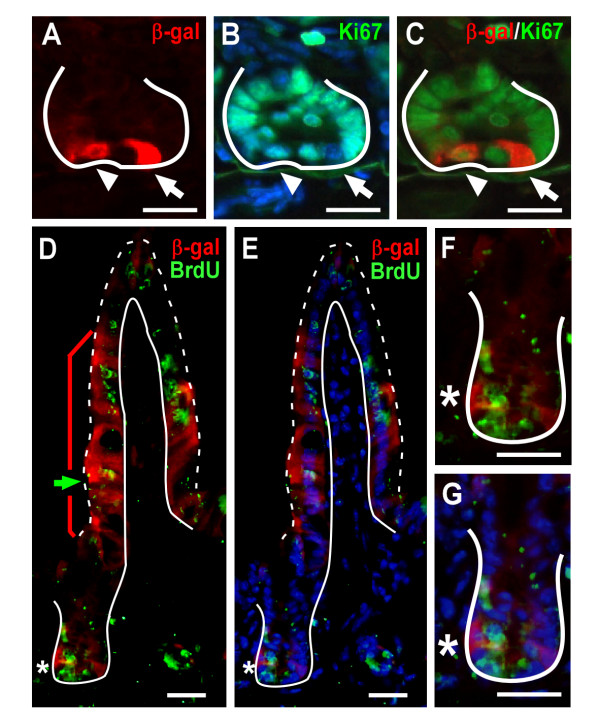
**Wnt-activated cells represent progenitor cells within the intestinal crypt**. (A-C) Cryopreserved intestinal tissue sections from TOPGAL adult mice co-stained with antibodies to β-gal (red) and Ki67 (green) then counter-stained with Hoechst (blue). Arrow indicates a cell with β-gal staining and arrowhead designates a cell co-staining for both markers. (D-G) Co-localization of BrdU (green) and β-gal (red) expression in crypt and villus epithelial cells from adult TOPGAL mice injected with BrdU 2 days prior to sacrifice. Green arrow denotes β-gal-positive cells at the lagging edge of migrating BrdU-positive cells up the villus. Red bracket indicates β-gal-positive villus epithelium. White asterisk marks β-gal and BrdU double-positive crypt cells. (F-G) Higher magnification of crypt regions in D-E. Solid white line demarks the epithelial-mesenchymal boundary. Dashed white line outlines the apical epithelial border. Counter-stained with Hoechst dye (blue). Bar = 25 μm.

To identify the intestinal epithelial cell population that actively receives a Wnt signal, we surveyed the entire length of the intestine from two independently established Wnt-reporter mouse lines, TOPGAL and BatGal[11,22], in addition to C57Bl/6 mice. TOPGAL and BatGal transgenic mice express the reporter, β-galactosidase (β-gal), in response to reception and processing of an endogenous canonical Wnt signal, marking cells activated by the signaling cascade[11,22]. Both mouse reporter lines displayed a similar pattern (Figure 1A, B), therefore TOPGAL mouse intestines are depicted unless otherwise noted. In the Wnt-reporter mouse intestine, we found strong β-gal expression in epithelial cells within the crypt base (Figure 1A, B, D). Typically, positive crypts within the proximal small intestine (PSI) contained only one or two β-gal-positive cells (Figure 1A–B), although some crypts were uniformly populated with β-gal-positive cells that extended into the TA-cell region (Figure 1C) and onto the adjacent villi. The reporter protein expression pattern was confirmed by detecting β-gal expression by enzymatic activity using the substrate, X-gal (Figure 1E). Interestingly, while single cells within the crypt base were detected, no crypts with the broader expression pattern, nor villus epithelial expression were observed. We corroborated our findings in the Wnt-reporter mice with identification of crypt cells harboring nuclear β-catenin in wild-type mice, a hallmark of Wnt activation (Figure 1F). The nuclear β-catenin staining pattern recapitulated the Wnt-reporter protein expression pattern (Figure 1A, B, D). Because of the discrepancy between the β-gal protein expression on the villus detected by antibodies (Figure 1C and 2D) and the crypt-based expression of β-gal enzymatic activity (Figure 1E), we analyzed reporter RNA expression. Both *in situ *hybridization (Figure 1G, H) and qRT-PCR for the *lac*Z gene in isolated crypt or villus epithelial populations (Figure 1I) demonstrated that Wnt-activated cells were restricted to the crypt base. An indepth examination of the crypt localization of β-gal-positive cells revealed that the majority resided in the base of the crypt (79.6%), the stem cell niche and the location of differentiated Paneth cells, while fewer β-gal-positive cells were located in the middle third (17.4%) or the upper third of the crypt (3.0%; Figure 1J).

Interestingly, a gradient of Wnt-activated, β-gal-positive cells existed in the intestine, with 15.2% of crypts containing a Wnt-activated cell in the PSI compared to 0.8% of colonic crypts (Figure 1K). Additionally, villi with β-gal-positive epithelium were also detected in a decreasing gradient down the length of the small intestine (Figure 1L). This pattern of Wnt-activated cells parallels the decreasing gradient in cell turnover and proliferation rates that exist down the length of the small intestine and colon[34].

### Wnt-receiving cells express cell proliferation markers but are not located in the TA-cell region

The majority of β-gal-positive cells reside in the base of the intestinal crypt, suggesting that Wnt signaling may influence proliferation in the progenitor population and not the TA-cell population. To distinguish if the Wnt signal conveys a restricted rather than global proliferative response within the intestinal crypt, intestines from Wnt-reporter mice were co-stained with antibodies to β-gal and the proliferation marker Ki67, which designates cells undergoing late G1, S, G2 or M phases of the cell cycle. Ki67-positive cells were located in the middle portion of the crypts and extended toward the lower third, consistent with the location of both the TA-cell population and crypt progenitor cells. Analysis was restricted to crypts containing one or two β-gal positive cells. In most of these crypts, the majority of the Ki67 staining did not co-localize with β-gal-positive cells (Figure 2A–C, arrow). Occasionally, β-gal-expressing crypt cells were also Ki67-positive (7.1%), potentially indicating that this Wnt-activated cell was actively dividing (Figure 2A–C, arrowhead).

**Figure 3:**
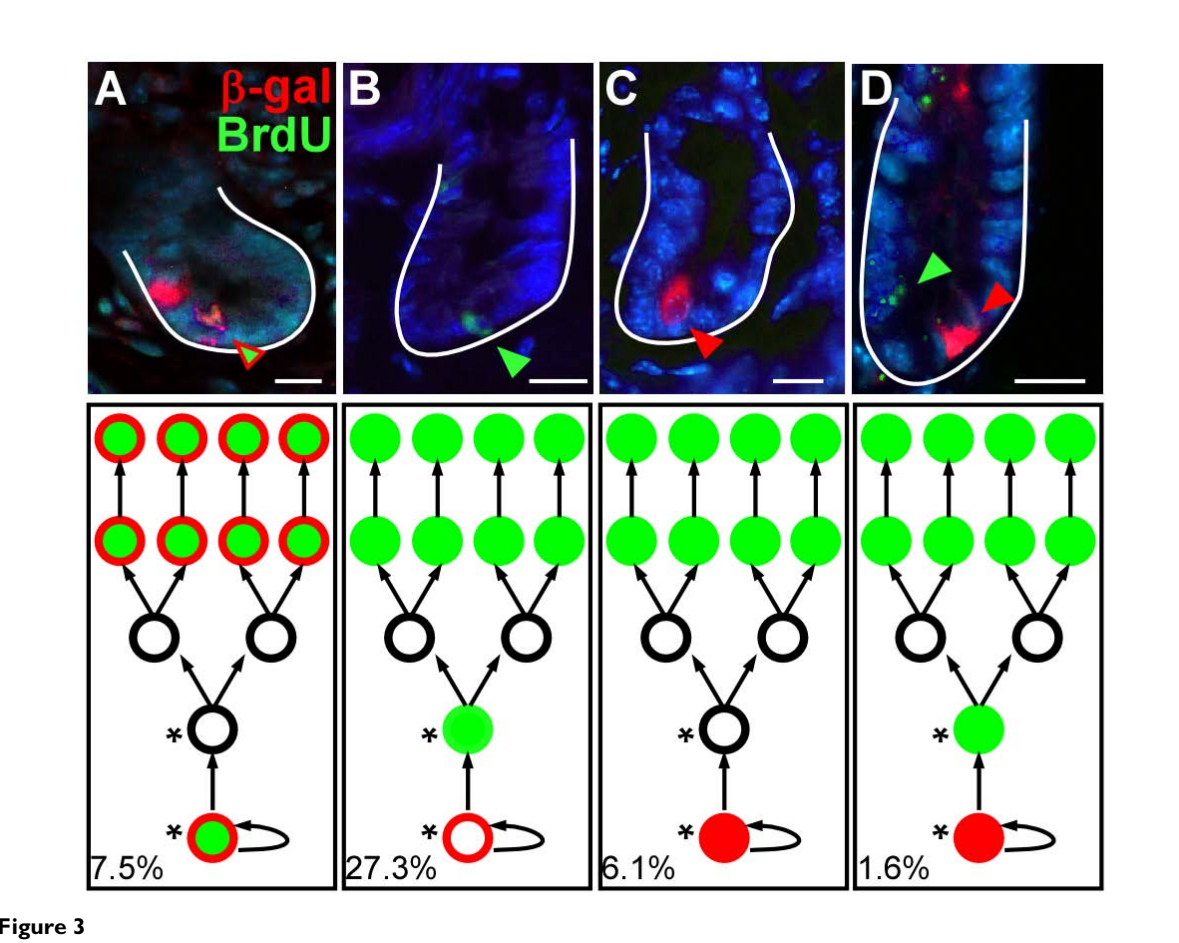
**β-gal and BrdU co-staining scenarios**. Wnt-reporter mouse intestines were injected with BrdU 2 days prior to analyses to assess the proliferative status of the β-gal positive crypt-based cells. (A) Approximately 7.5% of crypts contained a cell that was dual-labeled for β-gal and BrdU, reflecting cells that have been retained within the crypt (label-retaining cells) and that were Wnt-activated. (B) Approximately 27.3% of crypts contained a single BrdU-positive cell, possibly representing a "stem cell" that is not designated by the Wnt signaling pathway. This would be in line with the recently identified Bmi-1 positive stem cell. (C) 6.1% of crypts contained a single positive β-gal cell. This cell likely represents a cell that is activated by the Wnt signal after the effective BrdU labeling half-life in the animal. Finally, (D) a small percentage of crypts, 1.6%, contained a β-gal-positive cell and a BrdU-positive cell distinct from one another, likely representing a combination of the described scenarios. These scenarios are schematized in cartoon form beneath the corresponding fluorescent image that describes our perception of what each scenario may represent. In classical stem cell hierarchy, the lowest circle represents a progenitor cell residing near the base of the crypt and upper circles represent the progeny. Solid green circles represent BrdU-positive cells, solid red circles represent an activated Wnt cell, open red circles represent a cell that may have been Wnt-activated prior to BrdU labeling. These many different scenarios reflect the complex nature of the role of Wnt signaling on the stem cell hierarchy within the intestinal crypt.

To determine if the Wnt-activated cells were label-retaining cells, we performed BrdU label-retaining assays by injecting BrdU into TOPGAL mice 48 h prior to analysis. This timeframe is sufficient for BrdU-labeled epithelial cells to give rise to BrdU-positive descendents that have migrated up the villus (Figure 2D, lagging edge BrdU-cell marked by green arrow). At this analytical time point, the BrdU-labeled progeny have migrated away from the BrdU-label-retaining stem cell in the crypt (Figure 2D–G, white asterisk), as apparent by the intervening BrdU-negative cells (Figure 2D–E). The β-gal-positive villus epithelial cells overlap with the BrdU-positive villus cells (Figure 2D, red bracket) suggesting that they might be derived from the dual BrdU-positive, β-gal positive cell in the crypt. Approximately 7.5% of crypts contained cells with co-localized β-gal and BrdU (Figure 2F–G and Figure 3), suggesting that a subset of β-gal-positive cells were also crypt label-retaining cells.

**Figure 4:**
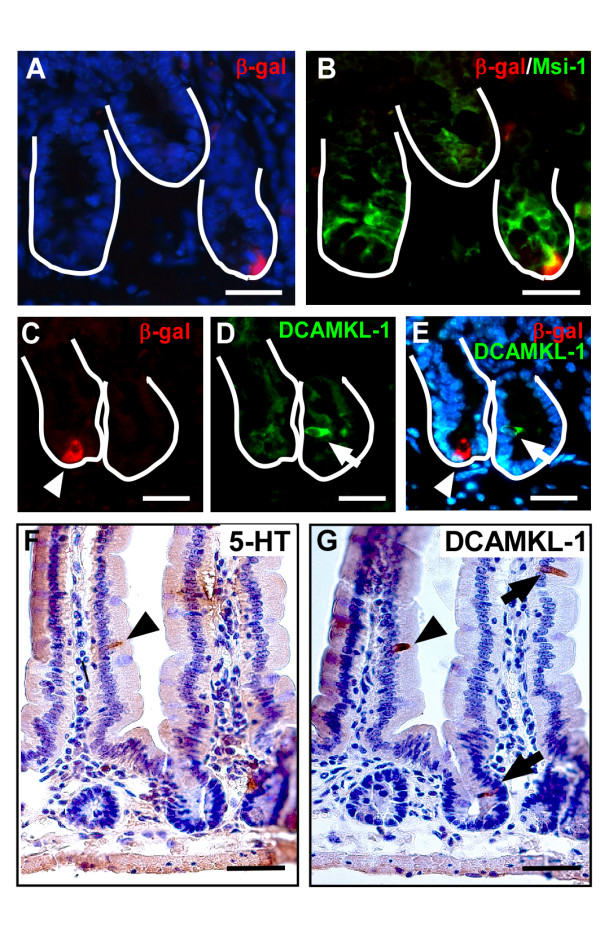
**Characterization of putative stem cell markers in Wnt-activated cells**. (A-B) The putative stem cell marker, Musashi-1 (Msi-1; green) had broad expression within the crypt and co-localized with crypt β-gal-expressing cells (red). (C-E) β-gal-positive cells (red) do not co-localize with another putative stem cell marker, DCAMKL-1 (green). Solid white line marks the epithelial-mesenchymal boundary of the intestinal crypt. (F-G) DCAMKL-1 is expressed in a subset of enteroendocrine cells. Serial sections of mouse PSI were stained for serotonin (5-HT, an enteroendocrine marker; F) or DCAMKL-1 (G), a proposed intestinal stem cell marker. Arrowheads mark a single cell that co-labeled with both antibodies. Arrows mark DCAMKL-1-positive cells that do not express serotonin. Bar = 25 μm.

To determine if Wnt-receiving crypt cells might share expression with stem or early progenitor cells, Wnt-reporter mouse intestines were stained with antibodies for a putative intestinal epithelial stem cell marker, Musashi-1 (Msi-1) [35-37]. Although Msi-1 and β-gal co-localized (Figure 4A–B), the Msi-1 antibody displayed a broader staining pattern within the crypt, also encompassing the TA-cell population. DCAMKL-1 is an alternative putative stem cell marker[38], however Wnt-activated cells did not co-express DCAMKL-1 (Figure 4C–E).

**Figure 5:**
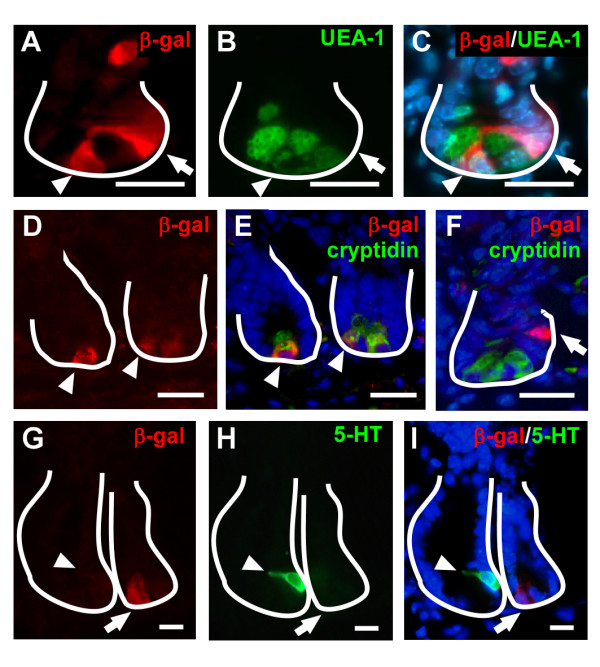
**Characterization of epithelial differentiation markers in Wnt-activated cells**. (A-C) Co-incubation of antibodies to β-gal (red) and UEA-1 (green), a lectin to mark Paneth and goblet cells, identifies distinct Wnt-activated cells (arrow) and overlapping expression (arrowhead). (D-F) Similar results are observed for the Paneth-cell-specific marker, cryptidin (green) when co-stained with β-gal (red). (G-I) Co-localization is not observed with dual staining of β-gal (red) and the enteroendocrine marker serotonin (5-HT; green). Solid white line marks the epithelial-mesenchymal boundary of the intestinal crypt. Bar = 25 μm.

Progenitor cell populations are not the only residents within the intestinal crypt. In the small intestine, differentiated Paneth cells reside at the crypt base, and differentiating goblet and enteroendocrine cells are also scattered within the crypt. Interestingly, a recent study implicated Wnt signaling in Paneth cell differentiation[39]. To determine if Ki67-negative/β-gal-positive cells were differentiated cells that resided at the crypt base[40], we stained Wnt-reporter mouse intestines with antibodies raised against epithelial differentiation markers. Co-localization of β-gal and the lectin, UEA-1, a dual goblet and Paneth cell marker, revealed that approximately 40.7% of the β-gal-positive cells possessed overlapping Paneth cell expression (Figure 5A–C; arrowhead), while 59.3% were distinct from Paneth cells (Figure 5A–C; arrow). Further, dual-labeling with antibodies to β-gal and cryptidin, a Paneth cell-specific marker, revealed similar findings (Figure 5D–F). It is possible that Wnt-activated, differentiated Paneth cells that retain β-gal protein are progeny from a Wnt-activated stem cell in a similar fashion as the β-gal-positive villus epithelial cells. Additionally, β-gal-positive cells were distinct from enteroendocrine cells when tissue sections were co-stained for the serotonin marker 5-HT (Figure 5G–I), and distinct from crypt-based goblet cells (data not shown).

### Wnt-reporter mouse intestine responded to physiologic increase in Wnt signaling

**Figure 6:**
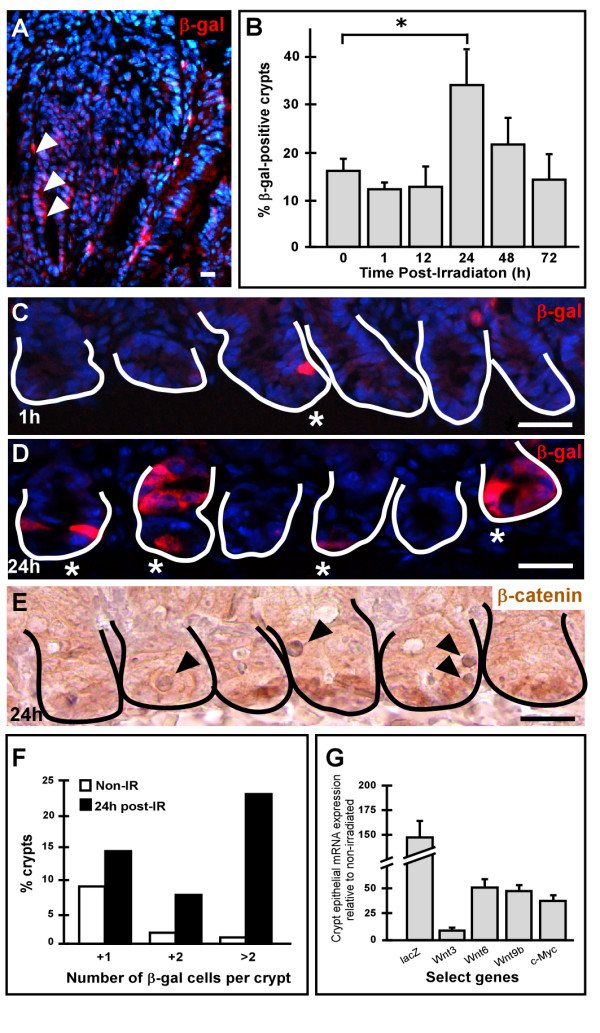
**Stimulation of Wnt signaling in intestinal epithelial cells**. (A) An increased number of Wnt-activated cells are detected in an intestinal adenoma from a progeny of a BatGal and Min mouse mating. β-gal-positive cells are in red (arrows). Wnt signaling is stimulated in response to gamma-irradiation-induced injury. (B) Intestinal tissue sections from lethally irradiated TOPGAL mice harvested at various timepoints were stained with antibodies to β-gal (red) and quantified. At 24 h post-irradiation, the number of crypts harboring Wnt-receiving cells significantly increased (*p *= 0.004; asterisk) relative to non-irradiated controls. (C-D) Comparison of representative intestinal tissue sections from lethally irradiated TOPGAL mice stained with antibodies to β-gal (red) and counterstained with Hoechst dye (blue) at 1 h post-irradiation (C) and 24 h post-irradiation (D). (E) Wild type mice, 24 h post-irradiation, were examined with antibodies to β-catenin (brown) and counterstained with Hematoxylin (purple). Solid line marks the epithelial-mesenchymal boundary of the intestinal crypts. At 1 h post-irradiation, the number of β-gal-expressing cells was similar to the 0 h control, but increased in 24 h post-irradiated tissues. Asterisks denote β-gal or nuclear β-catenin positive crypts; black arrowheads denote apoptotic cells. Bar = 25 μm. (F) The number of β-gal-positive cells per crypt was scored in both non-irradiated (Non-IR) and 24 h post-irradiated (post-IR) intestines. The percentage of crypts with 1, 2 or greater than 2 β-gal-positive cells are shown. (G) qRT-PCR performed on mRNA from small intestinal crypt fractions of TOPGAL mice 24 h post-irradiation revealed an increase in the *lacZ *reporter gene compared to non-irradiated samples. In addition, three Wnt ligands known to be expressed in the intestinal epithelium (*Wnt3*, *Wnt6*, and *Wnt9b*) and a Wnt target gene (*c-Myc*) increased in response to the irradiation stimulus.

In some intestinal diseases, such as colorectal cancer, the Wnt signaling pathway is aberrantly stimulated in epithelial cells resulting in uncontrolled hyperproliferation. This establishes a role for Wnt signaling in epithelial proliferation and highlights the importance of the Wnt signal in maintaining epithelial homeostasis. It has previously been shown, and we demonstrate here, that BatGal reporter mice display an increase in Wnt signaling readout when crossed to tumor-forming Min mice that overstimulate the Wnt pathway[22] (Figure 6A). To investigate if the Wnt-reporter mice are useful tools for increased Wnt signaling readout during tissue repair after injury, we examined the reporter response to intestinal gamma-irradiation exposure, which is known to stimulate an epithelial proliferative response.

Exposure to gamma-irradiation elicits massive crypt cell apoptosis, coincident with proliferative changes that peak within the first 24 hours [41]. To determine if activation of Wnt signaling is important in a regenerative response and if it can be monitored in a Wnt-reporter mouse, we subjected TOPGAL mice to 12 Gy of gamma-irradiation. Intestinal tissues were processed and analyzed 1, 12, 24, 48, and 72 h after irradiation (Figure 6B). At 1 h post-irradiation, the intestinal epithelium appeared relatively normal. Wnt-responsive cells, as detected by protein levels, were still present in low numbers in the crypts of the PSI (Figure 6B–C). However, by 24 h post-irradiation, near the peak of the apoptotic response, a significant increase in the number of crypts with Wnt-receiving cells was detected (*p *= 0.004, Figure 6B, D, E). Additionally, we observed more Wnt-receiving cells per crypt (Figure 6D–F) compared to non-irradiated controls (Figure 1A) or to the 1 h post-irradiation time point (Figure 6C). The most striking increase was represented by β-gal-positive crypts harboring greater than two Wnt-activated cells (Figure 6F). The Wnt response returned to non-irradiated, homeostatic levels by 72 h (Figure 6B).

Interestingly, the increase in Wnt-receiving cells paralleled an increase in β-gal/Ki67 double-positive cells (data not shown). While the majority of Ki67-positive cells remained β-gal-negative, dual β-gal and Ki67-positive cells increased approximately 3-fold (from 7.1% to 23.1%). This double-positive population may represent an actively dividing stem cell or immediate progeny from a newly divided progenitor cell.

To correlate increased Wnt responsiveness to gamma-irradiation, the mRNA expression levels of the three endogenous epithelial Wnt ligands were determined[33]. Crypt epithelium from 24 h post-irradiation and non-irradiated TOPGAL intestines was isolated and characterized for changes in Wnt ligand expression (Figure 6G). Reporter *lacZ *mRNA expression was elevated in response to gamma-irradiation exposure by ~148-fold in the crypt epithelium. Consistent with this observation, increased expression of the Wnt target gene *c-Myc *was observed (34-fold). Additionally, the canonical Wnt ligands *Wnt3*, *Wnt6*, and *Wnt9b *were also elevated by 10-, 51- and 50-fold respectively. Further, the mRNA expression of the secreted frizzled protein 2 (*sFrp2*), a Wnt inhibitor, decreased from levels higher than the Wnt ligands at steady state, to undetectable levels in response to gamma-irradiation (data not shown). This data suggests that induced injury to the epithelium results in detectable changes in Wnt signaling that can be appreciated in the Wnt-reporter mouse.

## Discussion

While it is well established that Wnt signaling controls intestinal epithelial proliferation and homeostasis, the distinction between the role of Wnt as a direct regulator of both the crypt-based stem cell and TA-cell populations has not been firmly established[1-3,5,6]. Further, aberrant Wnt signaling has been described as a proliferative stimulus in intestinal disease states such as colorectal cancer[5] but a role for the pathway in epithelial regeneration after injury has not been defined. Here we examined the pattern of Wnt-activated cells in the normal mouse intestine during homeostasis and after irradiation-induced injury. Further, we characterize intestinal expression of the Wnt-reporter mouse and show that it is a useful tool in both monitoring Wnt signaling during homeostasis and in response to an epithelial-induced injury.

In both TOPGAL and BatGal mouse intestines, Wnt-activated cells, as identified by Wnt-reporter expression, were primarily confined to the epithelial compartment. In the small intestine, two crypt-based patterns were observed. The majority of small intestinal crypts harbored one or two β-gal-positive cells detected by both protein and RNA localization. In a minority of crypts the entire crypt population was positive for β-gal protein expression that extended onto the adjacent villus. Additionally β-gal-positive cells were observed on villi that were associated with crypts containing single β-gal-positive cells. However, villus protein expression was not recapitulated with RNA expression profiling using *in situ *hybridization for *lacZ *on tissue sections or by qRT-PCR for *lacZ *expression in isolated crypt and villus epithelial cell populations. Together this suggests that Wnt-reporter expression on the villus was a manifestation of the long half-life of the β-gal protein[27]. The unique ability to track both protein and RNA expression in the Wnt-reporter mouse provides the power to analyze both lineage tracing (protein) and an identification of the Wnt-activated cell (RNA) within the same model system.

To corroborate that the Wnt reporter provided a consistent Wnt-activated cell readout, antibody staining to detect cells harboring nuclear localized β-catenin was performed. Consistent with the frequency of β-gal-positive cells, a similar percentage of PSI crypts harbored one or two cells near the crypt base that stained positive for nuclear β-catenin. These data suggest that only a small number of cells within certain crypts were actively receiving a Wnt signal.

Interestingly, but consistent with the observed decreasing gradient of epithelial cell turnover rates down the length of the intestine, a greater number of Wnt-activated cells were observed in the PSI (15.2% of crypts harbored at least one β-gal-positive cell) as compared to the colon (0.8%). Similarly, Bmi1-positive putative stem cells also display a gradient down the length of the intestine, with greater numbers in the PSI and nearly undetectable levels in the distal small intestine[42].

While it is widely accepted that Wnt signaling influences proliferation in all crypts, the number of Wnt-activiated cells detected in Wnt-reporter mouse intestines was lower than expected. There are several possibilities to explain this discrepancy. It is possible that the Wnt morphogen acts in a gradient highest in the base of the crypt and highest in the PSI with decreasing concentration down the length of the intestine. In this scenario, it is possible that only the highest levels of Wnt-activated cells are detected in the Wnt-reporter mice. Dilution of the protein as cells divide and migrate up the villus is therefore only detected in intestinal regions with the highest levels of Wnt activation. Presence of β-gal-positive villus cells may therefore identify regions of the intestine with robust Wnt signaling.

Some reports suggest a higher level of nuclear localized β-catenin in the crypt base than we show here[9,10]. Although believed to be a gold standard, comparing nuclear β-catenin with Wnt-activated cells could be misleading. Some cancer cells display high levels of nuclear β-catenin in the absence of Wnt activity [43]. The mechanism for this in cancer is unclear, although there are known inhibitors of nuclear localized β-catenin that inhibit Wnt activation by binding to β-catenin within the nucleus, including Apc, Chibby and Duplin [44-46].

Despite the decreasing gradient of detectable Wnt signal down the length of the intestine, there remains an important physiologic role of Wnt signaling in colonic homeostasis. It was recently reported that the Wnt target gene and putative stem cell marker, Lgr5, is located in base of both small intestinal and colonic crypts [8]. An alternative explanation for the proximal to distal gradient of detectable Wnt-activated cells could be that Wnt signaling in the colonic epithelium is regulated differently than in the small intestine. There are differences in expression of the Tcf/Lef-1 family members between the two regions[47] and therefore it is likely that other regulatory factors may convey differences in colonic Wnt activity. Due to these caveats in tracking Wnt-activated cells using other approaches, Wnt-reporter mice offer a powerful and direct approach for identifying Wnt-activated cells.

### Wnt-receiving intestinal cells represent a progenitor population

The rarity of single β-gal-positive and nuclear β-catenin-positive cells in the base of the crypt suggests that these Wnt-receiving cells may be a progenitor cell population. Therefore, to further characterize the proliferative status of the β-gal-positive cells, we surveyed intestinal sections with antibodies to Ki67 and β-gal. The majority of Ki67-positive cells were located mid-crypt in the TA-cell region and were negative for β-gal, thus not Wnt-activated. This suggests that Wnt signaling is not a general proliferative stimulant. Supportive of this observation, cells containing nuclear β-catenin were also not located within the proliferative TA-cell population, consistent with previous data from both the small intestine or colon[9,10]. Further, TA-cells have been shown to lack expression of a previously described Wnt-target gene, Lgr5, that marks a columnar crypt-based proposed stem cell[8]. This suggests that a second pathway may regulate proliferation of the TA-cell population. Recent evidence shows that the polycomb protein Bmi1, regulated in a Wnt-independent fashion, marks a putative intestinal stem cell population residing at "cell position 4" within the crypt[42]. Bmi1-expressing cells display a unique pattern from Lgr5-positive cells in the intestinal crypt[8]. These markers identify a population of "stem cells" with different kinetics, suggesting a more complex regulation of the intestinal stem cell hierarchy[48].

We observed that a portion of Ki67-positive cells were also β-gal positive. This represented 7.1% of all crypt-based β-gal positive cells and may possibly represent the stem cell or an early progenitor. We examined co-expression of β-gal with a putative stem cell marker, Msi-1. Even though the majority of β-gal-positive cells co-stained with this putative stem cell marker, Msi-1 displayed a broader pattern of expression that extended into the TA-cell region. While it is controversial whether or not Msi-1 is a true stem cell marker in the intestine, it may be expressed in a gradient including stem cells and their immediate descendents[35,37]. Despite this, co-localization of β-gal and Msi-1 supports the idea that Wnt-activated cells could represent progenitor cells. Interestingly, DCAMKL-1, a second putative stem cell marker[38], did not co-localize with β-gal positive cells. It is likely that DCAMKL-1 marks a lineage progenitor for enteroendocrine cells, as it is also expressed on the villus epithelium in a similar pattern with serotonin, an enteroendocrine cell marker (Figure 4F, G). Additionally, the putative stem cell marker, Lgr5, is reported to have an mRNA expression pattern encompassing a greater number of crypt cells and more total crypts[8] than the profile of Wnt-activated cells we show here. The overt discrepancy in staining patterns of the putative stem cell markers highlights the current dearth of tools available for pinpointing the intestinal stem cell *in vivo*.

We also observed a population of Ki67-negative, β-gal-positive cells. These cells might represent quiescent stem cells or the differentiated progeny of a Wnt-activated progenitor cell. Therefore, we performed double staining with β-gal and select antibodies for differentiated cell lineages. β-gal-positive cells did not express differentiation markers for goblet or enteroendocrine cells. Although a majority of the Paneth cells did not express β-gal (98.7%), a small subset was β-gal-positive. The presence of these double positive cells support the previously reported role for Wnt signaling in retaining Paneth cells to the crypt base[39]. Alternatively, these β-gal-positive Paneth cells could be recent descendents of an activated progenitor, as we show for differentiated epithelial cells (Figures 1C and 2D, E), highlighting the usefulness of protein detection for lineage tracing in this model system. Despite the role of Wnt signaling within the differentiated Paneth cell population, the majority of crypt-based β-gal-positive cells did not express differentiated cell markers (59.3%). Therefore, it is likely that these Wnt-activated cells represent a progenitor pool.

There is an emerging view of a more complex intestinal stem cell hierarchy with multiple pools of progenitor populations. In the absence of an intestinal reconstitution assay to validate Wnt-dependent and Wnt-independent putative stem cell pools, we cannot functionally determine the relationship of Wnt-activated cells within the hierarchy. It is likely that β-gal and nuclear β-catenin expression may be present in only a subset of stem cells. Additionally, quiescent stem cells might not express β-gal, nuclear β-catenin, Lgr5 or Bmi1. Despite these caveats, our data revealed a limited number of Wnt-activated cells within intestinal crypts and is consistent with a role for a Wnt signal in a progenitor pool.

### Wnt-reporter response to gamma-irradiation-induced injury

To determine if a Wnt signal was elicited in response to epithelial injury, we examined intestinal Wnt activation after gamma-irradiation. Upon exposure to gamma-irradiation, analyses of Wnt-reporter mice revealed an appreciable increase in both the number of crypts harboring Wnt-activated cells, as well as an increase in the total number of Wnt-activated cells per crypt. This observation was verified at the RNA level, demonstrating that irradiation-induced injury elicited an intestinal Wnt response. To confirm this increase in intestinal Wnt signaling, we surveyed for expression of a number of Wnt pathway genes in isolated epithelial crypt cells using qRT-PCR. An increase in *lacZ *was accompanied by increases in the three canonical Wnt ligands reported to be expressed in the crypt epithelium (*Wnt3*, *Wnt6*, *Wnt9b*) and the downstream target *c-Myc*. Further, a decrease in the secreted Wnt inhibitor (*sFrp2*) was observed. This demonstrated physiological intestinal damage can be appreciated using a Wnt-reporter mouse.

## Conclusion

Our data provides a carefully detailed analysis of endogenous Wnt signaling in the intestine of Wnt-reporter mice and corroborates reporter expression with nuclear β-catenin staining. Wnt-activated cells are predominantly located in the base of the crypt where a progenitor population and differentiated Paneth cells reside. This expression pattern is consistent with reported roles for Wnt signaling in maintaining a stem cell pool and in Paneth cell differentiation.

We demonstrate that the Wnt-reporter mouse can be used for *in vivo *analyses of both lineage tracing by detection of protein expression using immunohistochemistry and identification of Wnt-activated cell populations by reporter RNA expression. Importantly, our studies validate the use of the Wnt-reporter mouse (TOPGAL and BatGal) for detection of *in vivo *manipulation of Wnt signaling in response to intestinal epithelial injury.

## Abbreviations

β-gal: β-galactosidase; BrdU: 5-bromo-2-deoxyuridine; Gapdh: Glyceraldehyde-3-phosphate dehydrogenase; H&E: Hematoxylin & Eosin; Msi-1: Musashi-1; PSI: proximal small intestine; qRT-PCR: quantitative reverse transcriptase polymerase chain reaction; TA: transient-amplifying

## Competing interests

The authors declare that they have no competing interests.

## Authors' contributions

PSD and MHW participated in conception, execution of all experiments within the study, as well as the writing of the manuscript. ADD performed qRT-PCR analyses. AEP and KHC performed IHC analyses.

## Pre-publication history

The pre-publication history for this paper can be accessed here:


